# Pretraining neural and neuropsychological measures of executive functioning are associated with response to working memory training in Veterans with PTSD

**DOI:** 10.1017/S0033291726103298

**Published:** 2026-03-02

**Authors:** Christopher Hunt, Morgan Caudle, Amy Jak, Alan N. Simmons, Jessica Bomyea

**Affiliations:** 1 VA San Diego Healthcare System Center of Excellent for Stress and Mental Health, San Diego, CA, USA; 2 UCSD Department of Psychiatry, San Diego, CA, USA; 3 SDSU/UCSD Joint Doctoral Program in Clinical Psychology, San Diego, CA, USA

**Keywords:** cognitive training, executive functioning, post-traumatic stress disorder, trauma, working memory

## Abstract

**Background:**

Although there are several evidence-based treatments for post-traumatic stress disorder (PTSD), up to half of patients do not experience significant symptom relief. Executive functioning (EF) impairment is believed to impede PTSD recovery and diminish treatment response, but is not directly targeted by traditional treatments. Cognitive training for EF has emerged as a promising treatment alternative for PTSD, but may only benefit certain patients. The present study aimed to identify, validate, and characterize the subgroup of patients with PTSD who respond to an EF training program.

**Methods:**

Veterans with PTSD (N = 79) completed neuropsychological tests and a working memory task during functional magnetic resonance imaging scanning, followed by 16 sessions of an EF training program (working memory training [WMT]). Growth mixture modeling identified subgroups based on session-by-session working memory changes. Mixed-effects models then evaluated differences in spatial working memory and PTSD symptom improvement among these subgroups. Finally, the subgroups were compared on baseline neuropsychological performance and neural activity.

**Results:**

Three subgroups were extracted, with one subgroup (labeled low-WM/steep improvement subgroup) exhibiting steeper working memory improvement across training and greater spatial working memory and PTSD symptom improvement following training. The low-WM/steep improvement subgroup was uniquely characterized by a combination of lower EF task performance and lower working memory-related neural activity at baseline.

**Conclusions:**

WMT may be a promising alternative PTSD treatment for Veterans with EF impairments. Patients likely to benefit from WMT could be identified using a combination of neuropsychological and neuroimaging assessments, but further research is needed to confirm these indicators.

## Introduction

Although post-traumatic stress disorder (PTSD) can be addressed with a variety of treatments, even gold standard options fail to significantly improve symptoms in up to half of patients (Steenkamp, Litz, Hoge, & Marmar, 2015; Steenkamp, Litz, & Marmar, 2020). The heterogeneity of PTSD is becoming increasingly recognized as one impediment to treatment success (Cloitre, 2015; Herzog & Kaiser, 2022; Steenkamp & Litz, 2014). PTSD can exhibit a variety of symptom combinations, comorbidities, personality features, neurobiological correlates, and other characteristics (Morgan-Lopez et al., 2020; Young, Lareau, & Pierre, 2014), and at least some expressions appear to be partially undergirded by causes not well-suited to current PTSD treatments (Campbell, Trachik, Goldberg, & Simpson, 2020; Campbell-Sills et al., 2021). Developing alternative treatments for these differing presentations, and identifying the patients that best respond to them, could significantly increase the proportion of PTSD patients who experience symptom relief (Siegel & Laska, 2022). Such efforts are consistent with the broader goals of precision psychiatry to more effectively match patients to treatments tailored to their specific symptoms (Quinlan et al., 2020; Scala, Ganz, & Snyder, 2023).

One common PTSD correlate shown to impact treatment responsiveness is impaired executive functioning (EF). EF broadly refers to the ability to maintain control of complex goal-directed behavior (Alvarez & Emory, 2006; McCabe et al., 2010), and is typically measured by performance in areas like working memory, attention, response inhibition, flexibility, and planning (Aupperle, Melrose, Stein, & Paulus, 2012). Individuals with PTSD consistently exhibit lower EF compared to controls (Polak, Witteveen, Reitsma, & Olff, 2012; Woon et al., 2017). Moreover, impoverished EF is believed to exacerbate PTSD symptoms by disrupting the ability to disengage from trauma-related memories and reminders or inhibit the negative emotions they elicit (Aupperle et al., 2012; Bomyea & Lang, 2015). Neurobiologically, impoverished EF putatively reflects dysfunction in frontal areas necessary for PTSD recovery processes like fear extinction (Quirk, Garcia, & Gonzalez-Lima, 2006; Wicking et al., 2016) and emotion regulation (Andrewes & Jenkins, 2019; New et al., 2009). Importantly, first-line PTSD treatments do not directly address deficient EF (Crocker et al., 2018; Haaland, Sadek, Keller, & Castillo, 2016), which is among the strongest predictors of nonresponse to traditional trauma-focused therapies according to a recent meta-analysis (Keyan et al., 2024). Hence, PTSD treatments that directly target EF deficits may bring relief to a subgroup of patients that would not typically respond to standard interventions.

Cognitive training has emerged as an increasingly popular tool to enhance cognitive functioning (Jaeggi, Buschkuehl, Jonides, & Shah, 2011; Keshavan et al., 2014). Cognitive training for EF has been shown to effectively improve EF abilities in a variety of age groups (Ludyga et al., 2023; Nguyen, Murphy, & Andrews, 2019; Wass, 2015; Wass, Scerif, & Johnson, 2012) and in neurodevelopmental or neurocognitive disorders that may be marked by EF impairments (Chen et al., 2021; Pasqualotto et al., 2021; van de Ven, Murre, Veltman, & Schmand, 2016). To the extent that low EF impedes recovery from PTSD symptoms, intervening upon EF should improve PTSD symptoms. Indeed, several studies have found computerized EF training to produce greater PTSD symptom improvement than control or alternative training interventions (Badura-Brack et al., 2015; Bomyea, Stein, & Lang, 2015; Echiverri-Cohen et al., 2021). Additionally, EF training has been found to enhance the psychological processes theorized to mediate PTSD recovery (e.g. regulation of intrusive thoughts: [Bomyea & Amir, 2011]; emotion regulation: [Sabzi, Mihandoost, Nademi, & Parandin, 2022; Schweizer et al., 2013]) or their associated neural underpinnings (e.g. enhanced inferior frontal gyrus [IFG] activation: [Li et al., 2022]; increased IFG–amygdala connectivity: [Cohen et al., 2016]).

Although initial studies of EF training for PTSD have been promising, it is unclear which PTSD patients are most likely to benefit from this training. Recent evidence suggests that EF impairments are only present in a subgroup of PTSD patients (~15%) who also possess distinct neural characteristics and symptom trajectories relative to normal or high-EF PTSD patients (Jagger-Rickels et al., 2021; Jagger-Rickels et al., 2022). As such, EF training might only benefit the minority of PTSD patients who possess the neurocognitive deficits it targets. However, trials of EF training for PTSD have typically investigated the intervention in PTSD patients *as a whole* and not for the specific subgroup of patients whom the intervention might benefit most (Badura-Brack et al., 2015; Bomyea et al., 2015). Identifying the subgroup of patients who benefit most from EF training in terms of both cognitive and symptomatic improvement is a critical step toward maximizing the efficacy of this intervention for patients with PTSD.

Accordingly, the goal of the present study was to identify, validate, and characterize the subgroup of PTSD patients who respond to cognitive training for EF. Here, we examined data from a recently completed randomized clinical trial of Veterans with PTSD undergoing working memory training (WMT). WMT is a commonly used method to enhance EF (Karbach & Verhaeghen, 2014; Morrison & Chein, 2011; Salminen, Strobach, & Schubert, 2012) and is particularly relevant to PTSD given that concentration issues (which reflects poor WM) are a PTSD symptom (APA, 2013) and because deficits in WM systems are thought to influence management of other PTSD symptoms (Norte, Vargas, & de Carvalho Silveira, 2022). PTSD subjects also tend to exhibit lower working memory capacity (Honzel, Justus, & Swick, 2014; Nejati, Salehinejad, & Sabayee, 2018) and be more affected by emotional distractors during WM tasks (Morey et al., 2009; Schweizer & Dalgleish, 2016), which putatively makes detaching from trauma-related memories and emotions more difficult. There is also preliminary evidence that training-related improvements in WM lead to improvements in PTSD symptoms (Bomyea & Amir, 2011; Larsen et al., 2019), thus further supporting the role of WM deficits in maintaining PTSD symptoms.

In this study, we specifically investigated (a) whether there was a distinct subgroup who responded to WMT based on improved working memory scores across training (i.e. identification); (b) whether this group also showed improvements in a non-training working memory task and in PTSD symptoms across training (i.e. external validation); and (c) whether this group could be adequately identified with baseline demographic, behavioral, and neural data (i.e. characterization). We hypothesized that there would be a distinct subgroup of PTSD patients who exhibit improved working memory and PTSD symptoms across training and further hypothesized that this subgroup would be characterized by low working memory scores and attenuated activation in working memory-related neural areas at baseline, consistent with WMT working best for patients with deficiencies in its putative treatment target.

## Materials and methods

### Participants

Participants were Veterans recruited from the VA San Diego Healthcare System. Qualifying participants were aged 21–65 with a current PTSD diagnosis, no history of severe mental illness (e.g. psychosis) or traumatic brain injury, and no recent severe substance use or suicidality, among other qualifications (see Supplementary Section 2.1 for full inclusion/exclusion criteria). *N* = 79 participants were eligible, enrolled, and attended the minimum number of training sessions (2). Of these 79 participants, *n* = 45 attended the functional magnetic resonance imaging (fMRI) session and had usable fMRI data while *n* = 56 completed the full neuropsychological battery at pretreatment. Of note, the relatively lower sample size for the fMRI scan and neuropsychological assessment was due to the study overlapping with the COVID-19 pandemic for two years, which prevented access to these in-person visits. Full information on participant flow can be found in the CONSORT diagram (Figure 1S of the Supplementary Material) and in Section 2.1 of the Supplementary Material. Demographics and clinical characteristics of the final sample can be found in Table 1S of the Supplementary Material.

### Procedure

Following an initial eligibility screening, participants completed a baseline intake appointment where they completed self-report scales, neuropsychological tasks, and interviews to confirm full eligibility (see Supplementary Section 2.2 for specifics), and an fMRI scan. Following the baseline assessment, participants were assigned to 16 sessions of WMT occurring twice weekly for eight weeks. Each WMT session involved repeated completion of a modified complex reading span (R-SPAN) task, which required the participant to remember presented stimuli while doing a secondary problem-solving task. Participants were randomized to one of two WMT conditions: low interference control (LIC), which included both letter and number stimuli, and high interference control (HIC), which included only letters, thereby increasing proactive interference during recall (see Bomyea et al., 2015 and Supplementary Section 2.3 for more details about WMT protocol and LIC/HIC conditions). Differences between LIC and HIC groups were not examined in this study since (a) our hypotheses were about WMT in general and not interference control specifically, (b) the LIC and HIC were identical in all other key parameters related to WMT (e.g. identical stimulus spans), and (c) because the identified WMT subgroups (see Results Section ‘WMT subgroup identification’) were already relatively small (lowest *n* = 16) and further dividing analyzed cells into treatment conditions would further reduce statistical power. However, we did examine differences in LIC/HIC randomization in the identified subgroups to ensure such subgroups were not merely a recapitulation of treatment conditions. A brief set of self-report questionnaires, including an assessment of PTSD symptoms (PCL-5), was conducted midway through treatment (i.e. following session 8) and at the conclusion of treatment (i.e. following session 16). Participants also completed the same working memory assessments from baseline at posttreatment. All participants provided written informed consent, and the trial protocol was approved by the VA San Diego Institutional Review Board. The authors assert that all procedures contributing to this work comply with the ethical standards of the relevant national and institutional committees on human experimentation and with the Helsinki Declaration of 1975, as revised in 2008.

### Measures

#### Working memory

Changes in working memory across treatment were assessed with the modified R-SPAN task used to train working memory. The R-SPAN task instructs participants to remember stimuli while simultaneously completing a processing task. At the start of each trial, a fixation cross appears for 500 ms. Next, the screen displays a processing task (e.g. ‘Jane walks her car in the park’). After the processing task, the screen displays a question about the task (e.g. ‘does this sentence make sense?’) and prompts the participant to choose ‘True’ or ‘False’. The processing task is followed by the presentation of a to-be-remembered-item for 500 ms. The subsequent trials follow the same pattern – the presentation of a processing task followed by an item. At the end of a set, a recognition screen is displayed with twelve items and participants were instructed to choose and arrange previously displayed to-be-remembered items in serial order. Then, new trials began and followed the same pattern. Sets contain between two and six trials (i.e. between 2 and 6 to-be-remembered items out of the 12 total items presented on the recognition screen). Each session contained three blocks of 15 sets. The scanner R-SPAN task was identical to the treatment R-SPAN except that it included sets of two, four, or six letters or numbers, with each set size being repeated seven times in pseudorandom order Correct responses were separately tabulated for each of the three runs during the session (range per run = 0–60), and the highest R-SPAN total served as the participant’s score for that session as a reflection of maximum working memory ability (for a detailed description of this task see Supplementary Sections 2.3.2. and 2.4.3.2).

#### PTSD symptoms

Changes in PTSD symptoms were captured using the PTSD Checklist for DSM-5 (PCL-5; Blevins et al., 2015), which was administered at baseline, mid-treatment, and posttreatment.

#### Neurocognition

Identified WMT subgroups were compared on both neurocognitive measures meant to assess EF and clinical and demographic variables that might affect cognition. Neurocognitive baseline variables related to EF included: verbal working memory ability (R-SPAN) (Bomyea, Taylor, Spadoni, & Simmons, 2018), spatial working memory ability (symmetry span [S-SPAN]; Unsworth, 2010), cognitive flexibility (letter-number switching raw score from Delis–Kaplan EF System Trail Making Test [D-KEFS TMT]; Delis, Kaplan, & Kramer, 2001), and response inhibition (switching-inhibition raw score from the D-KEFS color word interference test [D-KEFS CWI]). We also assessed two processing speed variables – visual scanning from the D-KEFS TMT and word reading from the D-KEFS CWI – to assess whether differences between WMT subgroups on EF variables were specific to EF or related to cognition more generally.

#### Clinical and demographic variables

Clinical baseline variables included: insomnia (insomnia severity index; Morin, Belleville, Bélanger, & Ivers, 2011), depression (Beck Depression Inventory-II [BDI-II]; Beck, Steer, & Brown, 1996), time (in months) since trauma, and TBI history classification using established TBI guidelines (O’Neil et al., 2013). We also examined age differences between WMT subgroups given its potential role in neurocognition, and number of sessions attended to assess whether differences in response to training were driven by engagement in training.

#### Neural data

Participants were scanned in a 3-Tesla GE 750 scanner using a 32-channel head array coil. The scanning session included collection of a three-plane scout scan, of a sagittally acquired sequence for acquiring T1-weighted images (parameters: FOV 256 mm; matrix: acquisition matrix =256 × 256 with 0 mm gap; 208 slices; thickness: 1 mm; TR 6900 ms, TE = 2.9 ms, flip angle: 8°) and one T2*-weighted axially acquired multiband echo-planar imaging scan to measure blood oxygen level dependent (parameters: FOV = 216 cm, acquisition matrix = 90 × 90 with a 0 mm gap; TR = 800 ms; TE = 30 ms; flip angle = 52° [whole brain]).

### Statistical analyses

WMT subgroups were identified using growth mixture modeling (GMM), a technique used to identify unobserved subsamples in a dataset based on differences in longitudinal change on one or more outcomes (Ram & Grimm, 2009). GMMs were run using the hlme command within the lcmm package in R (Proust-Lima, Philipps, & Liquet, 2017). Session-by-session peak R-SPAN scores served as the dependent variable while session number (1–16) served as the within-subject predictor variable. Bayesian Information Criterion (BIC) values were examined for model fit. We first identified the best-fitting (lowest BIC) model in terms of the number of latent subgroups (between 1 and 4), random effects (none, intercept, or intercept and slope) and slope shapes (linear and logarithmic). The best-fitting model was then selected for interpretation of subgroup-specific intercepts and slopes (see Supplementary Section 2.4.1 for more information). To ensure generalizability of the solution, we also reran the model including only participants who completed the baseline neuropsychological assessment (*n* = 56).

For subgroup validation, we utilized mixed-effects models (MEMs) to test whether the extracted GMM subgroups exhibited different patterns of change on two exogenous outcomes of interest: spatial working memory (S-SPAN) and PTSD symptoms (PCL-5). We examined changes in S-SPAN scores across WMT subgroups to verify that subgroup differences on the training task (R-SPAN) generalized to a working memory task from a different modality (S-SPAN). Changes in PCL-5 scores between WMT subgroups tested whether group differences in WM improvement translated into differences in PTSD improvement. We also repeated the same model with BDI-II scores to verify that any significant results for PCL-5 were specific to PTSD and not shared with depression. Each MEM included WMT subgroup, time, and their interaction, which tested whether the extracted subgroups showed different slopes of spatial working memory change and PTSD change across training. Random effects (intercept and slope) and slope shapes (linear vs. logarithmic) were selected according to how much they lowered BIC values. S-SPAN was only measured at pre- and post-WMT so logarithmic slopes could not be tested. Since WMT subgroup was a categorical variable, MEMs were tested with contrast coding in which the group with the greatest R-SPAN improvement during training was treated as the reference group. A significant Group × Time interaction for PCL-5 total was followed up by examining the same model for the four PCL-5 subscales. For PCL-5 subscale models, we applied a Benjamini–Hochberg (BH) correction for multiple comparisons (Benjamini & Hochberg, 1995), wherein *p* values are ranked from smallest to largest and evaluated at a threshold of *α*×(*I*/*M*), where *α* is the original significance threshold (.05), *I* is the *p* value ranking, and *M* is the total number of tests.

For subgroup characterization, we compared the WMT subgroups on baseline variables of interest (see Section ‘Measures’ for full lists) using separate one-way ANOVAs and a BH correction for multiple comparisons. Chi square tests were used to examine differences between WMT subgroups in randomization to LIC versus HIC, since this test involved count data.

Neural data were processed utilizing Analysis of Functional NeuroImages and Advanced Normalization Tools Software in R (see Supplementary [Section: 2.4.3.2 Neural data] for a detailed description of fMRI data processing pipeline and control for multiple corrections). To analyze neural functioning during the R-SPAN task, response regressors were created for each phase of the task. Three phases were determined by the unique timing of presentation for each phase for each participant, including sentence reading and verification, stimuli encoding, and stimuli recall. Neural baseline differences between WMT subgroups were analyzed using a 3dLME of the R-SPAN task encoding phase weighted according to set size (i.e. the number of stimuli presented in a single trial), within an a priori defined working memory mask using neuroanatomical atlases of regions relevant to working memory (NeuroSynth v5 ‘working memory’ Topic 045). To further evaluate the clinical significance of these regions, extractions from each ROI were used as predictors of PTSD symptom changes across training using an identical MEM as was used to test the effects of WMT subgroup. These tests were also subject to BH correction for multiple comparisons based on the number of tested ROIs.

## Results

### WMT subgroup identification

**Figure 1.**
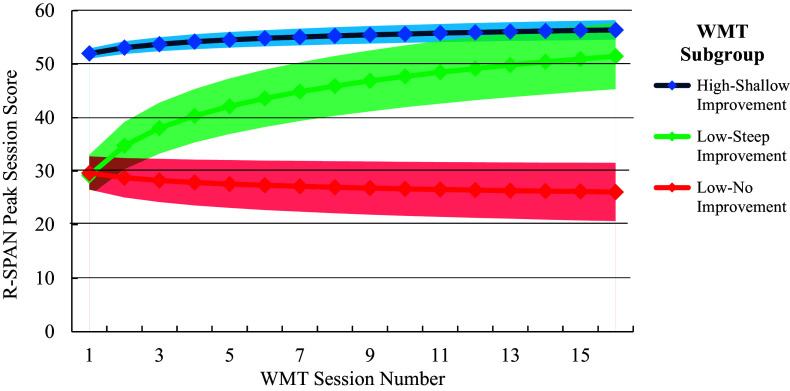
Fitted value plot of working memory trajectories across WMT sessions for each GMM subgroup. Shaded areas represent the 95% CI of the trajectory. R-SPAN highest total score is the highest R-SPAN score among the three R-SPAN runs that were completed at each session. Subgroups were extracted from the best-fitting GMM of session-by-session highest R-SPAN scores across the 16 WMT sessions. Note: WMT, ‘Working memory training’; R-SPAN, ‘Reading Span’; GMM, ‘Growth mixture model’; 95% CI, ‘95% confidence interval’.

The best-fitting model included a random intercept and slope, a logarithmic slope shape, and three latent subgroups (see Table 2S for full model statistics). The latent subgroups extracted from the best-fitting model included: (1) a high-WM/shallow improvement subgroup (54.5% of the total sample), characterized by a high intercept (*b* = 51.97, 95% CI = [50.03, 53.91], *p* < .001) and a shallow but significant positive slope (*b* = 1.59, 95% CI = [0.97, 2.21), *p* <. 001); (2) a low-WM/steep improvement subgroup (20.3% of the sample), characterized by a low intercept (*b* = 29.21, 95% CI = [22.99, 35.43], *p* < .001) and a steep positive slope (*b* = 8.03, 95% CI = [6.35, 9.71], *p* < .001); and (3) and a low-WM/no improvement subgroup (25.2% of the sample), characterized by a low intercept (*b* = 29.65, 95% CI = [22.01, 37.29], *p* < .001) and a nonsignificant negative slope (b = −1.28, 95% CI = [−2.98, 0.42], *p* = .131). Rerunning the analysis including only those who completed the baseline neuropsychological assessment yielded virtually identical findings, including the same best-fitting model and the same subgroup parameters (see Supplementary Table 2S for complete statistics). A fitted value plot of session-by-session R-SPAN score changes for each WMT subgroup can be found in Figure 1.

### WMT subgroup validation

**Figure 2.**
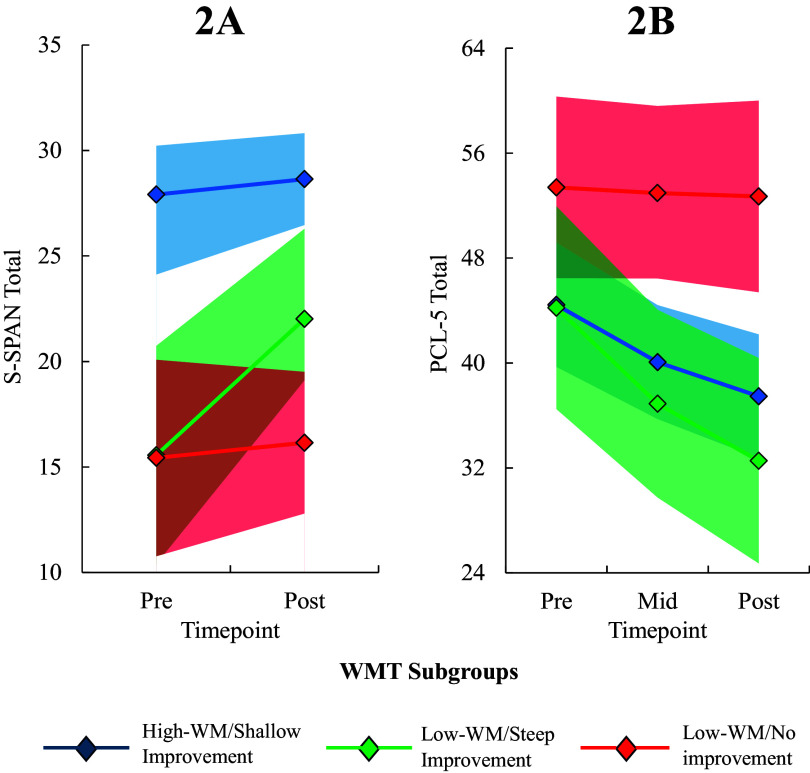
Fitted value plot of (a) spatial working memory [S-SPAN] and (b) PTSD symptoms [PCL-5] across WMT for each WMT subgroup. Shaded areas represent the 95% CI of the trajectory. Subgroups were extracted from the best-fitting GMM of session-by-session highest R-SPAN scores across the 16 WMT sessions. Note: S-SPAN, ‘Symmetry span’; PCL-5, ‘PTSD Checklist for DSM-5’; WMT, ‘Working memory training’; R-SPAN, ‘Reading Span’; GMM, ‘Growth mixture model’; 95% CI, ‘95% confidence interval’.

The S-SPAN MEM revealed a significant interaction between time (pre- and post-training) and WMT subgroup, *F* = 3.28, *p* = .044, which follow-up analyses revealed was driven by significantly steeper improvement in the low-WM/steep improvement subgroup relative to both the low-WM/no improvement subgroup (*b* = −3.20, 95% CI = −6.05, −0.35, *p = .*029) and the high-WM/shallow improvement subgroup (*b* = −2.83, 95% CI = −5.22, −0.44, *p* = .021). Thus, the low-WM/steep improvement subgroup exhibited superior working memory improvement across training relative to the other two subgroups on both the verbal WMT task and a non-training spatial working memory task (see Figure 2 for fitted value plots).

The PCL-5 MEM was best characterized with a logarithmic slope and the Log time × WMT subgroup interaction was significant at the level of a trend (*p* = .056). Follow-up analyses revealed that the low-WM/steep improvement subgroup exhibited a significantly steeper reduction in PCL-5 scores relative to the low-WM/no improvement subgroup (*b* = 10.00, 95% CI = 1.77, 18.21, *p* = .018) but not the high-WM/shallow improvement subgroup (*b* = 4.28, 95% CI = −2.68, 11.23, *p* = .223). Nonetheless, the overall pattern of results suggested that subgroups with greater working memory improvement showed greater PTSD improvement. In contrast, none of the Group × Time interactions for the PCL-5 subscales reached statistical significance after applying a BH correction for multiple tests (*p* > .018). Similarly, no significant WMT Subgroup × Log Session interactions were found for BDI-II, indicating changes in depression symptoms across WMT did not differ significantly between subgroups (*p*s > .130). Comparison of model fit statistics for each candidate MEM can be found in Table 3S of the Supplementary Material. Coefficients in the best-fitting MEMs used to predict changes in S-SPAN and PCL-5 from WMT subgroup can be found in Table 4S of the Supplementary Material.

### WMT subgroup characterization

#### Self-report and neuropsychological data

One-way ANOVAs revealed that WMT subgroups differed on all EF-related measures at baseline, including R-SPAN, *F* = 37.30, η^2^ = 0.50, *p* < .001, S-SPAN, *F* = 20.03; η^2^ = 0.35, *p* < .001, TMT switching, *F* = 8.65, η^2^ = 0.25, *p* < .001, and CWI inhibition/switching, *F* = 9.20, η^2^ = 0.26, *p* < .001. Post hoc pairwise comparisons revealed that these differences were, in almost all cases, driven by the high-WM/shallow improvement subgroup scoring significantly higher than the other two subgroups (*p*s < .021), which not differ from each other (*ps* > .559). The lone exception was that the high-WM/shallow improvement and low-WM/steep improvement subgroups did not significantly differ on TMT switching (*p* = .090). No other differences in baseline variables between subgroups emerged after correcting for multiple comparisons (*p*s > .030). Full statistics for all ANOVAs comparing subgroups on baseline measures can be found in Table 1.

**Table 1.** Differences between WMT subgroups on demographics and baseline variables

Measure	High-WM/Shallow Improvement Subgroup mean (SD) or %	Low-WM/Steep Improvement Subgroup Mean (SD) or %	Low-WM /No Improvement Subgroup Mean (SD) or %	F or χ^2^	η^2^ or V	*p*
Age	38.26 (8.76)	36.25 (8.55)	40.81 (8.59)	1.23	0.03	.298
Education	4.05 (1.69)	4.20 (1.70)	4.60 (1.35)	0.64	0.02	.532
PCL–5 total	42.91 (14.44)	44.81 (15.35)	53.50 (13.98)	3.68	0.09	.030
Months since trauma	140.44 (116.93)	162.63 (100.56)	118.26 (74.96)	0.78	0.02	.461
ISI total	15.23 (5.59)	18.15 (5.65)	15.19 (6.40)	1.92	0.05	.153
BDI-II total	24.19 (11.60)	30.50 (9.02)	26.06 (10.04)	2.38	0.06	.100
TBI severity	0.93 (0.75)	1.00 (0.73)	0.81 (0.83)	0.27	0.01	.765
HIC %^a^	51.2	50.0	60.0	0.61	0.09	.738
Number training sessions	14.30 (4.35)	14.07 (4.42)	12.55 (4.65)	1.09	0.03	.340
R-SPAN total	63.83 (12.00)	37.33 (14.80)	43.07 (15.20)	37.30	0.50	<.001
S-SPAN total	27.91 (7.35)	15.79 (7.27)	19.80 (7.48)	20.03	0.35	<.001
TMT scanning^b^	19.23 (3.91)	22.29 (6.33)	19.67 (5.56)	1.86	0.07	.165
TMT switching^b^	62.87 (19.59)	74.83 (21.64)	97.62 (37.30)	8.65	0.25	<.001
CWI color naming^b^	28.27 (4.95)	33.86 (7.35)	33.33 (12.61)	3.35	0.11	.043
CWI inhibit/switch^b^	52.23 (10.01)	75.86 (29.79)	69.92 (18.34)	9.20	0.26	<.001

*Note:* Education was self-reported on a 7-point ordinal scale spanning 1 (did not finish high school) to 7 (graduate degree). TBI severity levels were computed from TBI history questions and ranged from 0 (none) to 3 (severe). WMT subgroups were formed based on differences in working memory changes across WMT. Comparisons were evaluated at a BH-corrected significance threshold based on the number tested covariates (14).

Abbreviations: WMT, Working memory training; PCL-5, PTSD Checklist for DSM-5; ISI, Insomnia Severity Index; BDI-II, Beck Depression Inventory II; TBI, Traumatic brain injury; R-SPAN, Reading span; S-SPAN, Symmetry span; TMT Scanning, Trail Making Test visual scanning subset; TMT Switching, Trail Making Test letter-number switching subtest; CWI Color Naming, Color Word Interference color naming subtest; CWI Inhibit/Switch, Color Word Interference, Inhibition/Switching subtest. *N* = 79.

a Categorical variable tested with chi-squared (χ^2^ and V statistics).

b Measure available for only a subset of participants (*n* = 56).

Overall, baseline measures of neuropsychological functioning were able to successfully distinguish the high-WM/shallow improvement group from the other two subgroups (i.e. higher EF in the high-WM/shallow improvement subgroup) but were not able to distinguish the other two subgroups (low-WM/steep improvement and low-WM/no improvement subgroups) from each other. Because the high-WM/shallow improvement could be readily distinguished from the other two subgroups using neuropsychological testing, we did not include this subgroup in our analyses of neural differences, as fMRI is only clinically justifiable in cases in which more accessible measures fail to yield phenotypic differences.

#### Neuroimaging data

**Figure 3.**
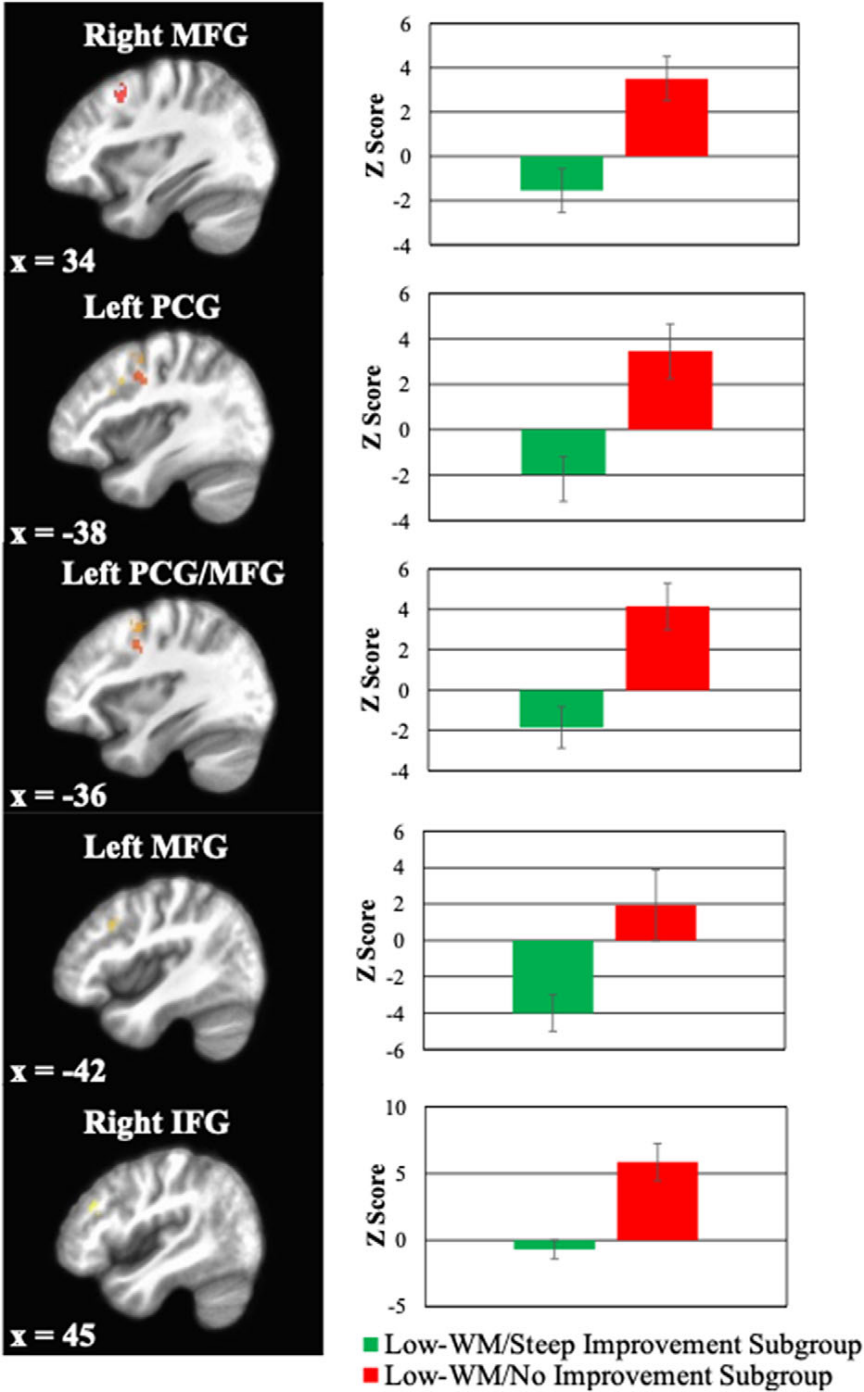
Standardized estimates of neural activation (z-score) in the right middle frontal gyrus (MFG), left precentral gyrus (PCG), left PCG extending into left MFG, the left MFG, and the right inferior frontal gyrus (IFG) during the stimulus encoding portion of the baseline R-SPAN task. Bars represent activation in the low-WM/steep improvement subgroup (blue) relative to the low-WM/no improvement subgroup (orange).

Within the a priori defined working memory mask, the low-WM/steep improvement subgroup exhibited significantly lower neural activity during R-SPAN encoding in five ROIs relative to the low-WM/no improvement subgroup (*p*s < .004; see Figure 3 for images of neural regions and Table 5S for coordinates). These ROIs spanned the bilateral middle frontal gyrus (MFG), left precentral gyrus (PCG), left PCG extending into the left MFG, and the left IFG. To verify that this pattern of low neural responding in WM regions was unique to the low-WM/steep improvement subgroup, we conducted a supplemental analysis comparing the low-WM/steep improvement subgroup to both other subgroups. This analysis revealed differences in 13 ROIs (see Table 6S for full list and coordinates), with five of seven largest ROIs being the same as those found in the two-group analysis (i.e. bilateral MFG, left PCG, left PCG extending into the left MFG, and left IFG). Pairwise comparisons for these regions again showed lower activation in the low-WM/steep improvement subgroup compared to the other two subgroups, which did not differ from each other (see Figure S3 of the Supplement for an illustration of subgroup differences). Hence, findings from the three-group analysis confirmed that lower neural activation was unique to the low-WM/steep improvement subgroup.

**Figure 4.**
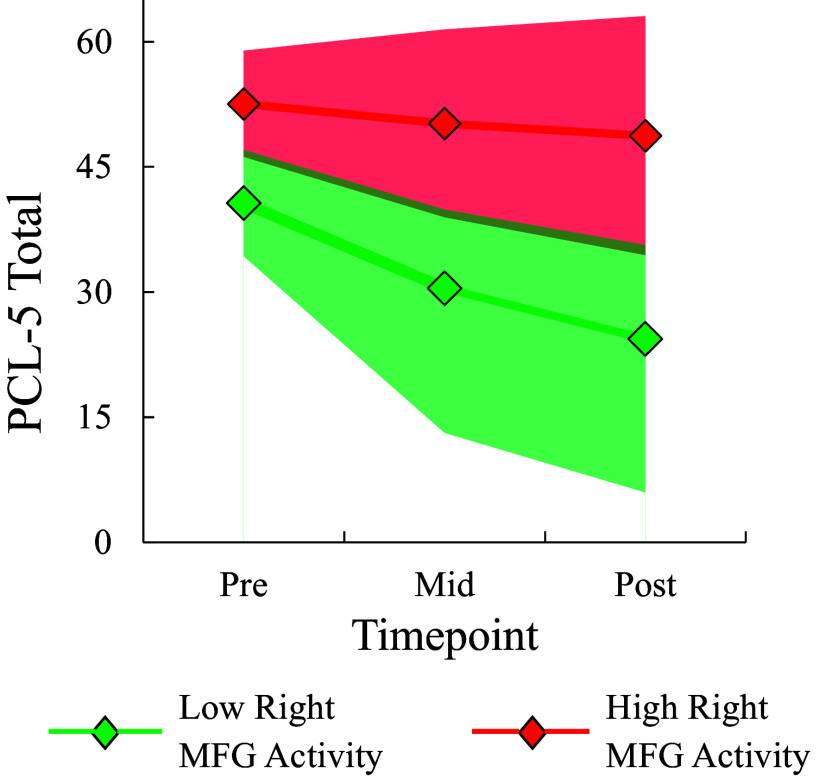
Fitted value plot of PCL-5 trajectories across pre-, mid-, and post-WMT for participants exhibiting high versus low baseline neural activity in the right MFG at baseline. Shaded areas represent the 95% CI of the trajectory. Models were evaluated in the WMT low-WM/no improvement and low-WM/steep improvement subgroups only. Neural activity was measured during encoding phase of the R-SPAN task at baseline. High and low activity reflect estimated neural activation at +1SD and −1SD below the mean, respectively. Note: PCL-5, ‘PTSD Checklist for DSM-5’; WMT, ‘Working memory training’; MFG, ‘Middle frontal gyrus’; 95% CI, ‘95% confidence interval’; SD, ‘standard deviation’.

MEMs predicting PCL-5 changes revealed significant interactions with log time for activity in each ROI (*p*s < .026; see Table 7S of the Supplementary Material for full statistics). In each case, lower activity in the ROI predicted a steeper reduction in PCL-5 scores across WMT. A fitted value plot depicting PCL-5 changes across training for participants with high versus low activity in the largest ROI (right MFG) can be found in Figure 4. Since effects were similar for the other ROIs, their corresponding fitted value plots were not constructed.

## Discussion

The purpose of this study was to identify, validate, and characterize the subgroup of PTSD patients who respond to WMT. Results confirmed the existence of a distinct subgroup of patients who exhibited steep improvement in working memory across training, as well as two less responsive subgroups – one group that began with high working memory and showed only modest improvement and another group that began with low working memory and showed no improvement. The low-WM/steep improvement subgroup also exhibited greater improvement in spatial working memory compared to the other two subgroups and greater improvement in PTSD symptom improvement relative to the low-WM/no improvement subgroup. At baseline, the low-WM/steep improvement subgroup could be distinguished from the high-WM/shallow improvement subgroup by relatively lower EF across most baseline measures and from the low-WM/no improvement subgroup by lower activity in brain areas linked to working memory (MFG, left PCG, left IFG), which also predicted steeper PTSD improvement across training. Overall, these findings highlight the potential of WMT to improve working memory and PTSD symptoms in a distinct minority of patients who exhibit lower EF and lower working memory-related neural activity prior to training.

Findings from the present study are broadly consistent with an experimental therapeutics model in which a treatment’s efficacy is maximized when it is delivered to patients with the deficit it is designed to target (Gordon, 2017). Patients who responded best to WMT generally exhibited lower baseline levels of the target process on both a neurocognitive level (lower EF performance) *and* a neural level (lower activity in brain areas considered integral to working memory). Although the subgroup with superior baseline EF (high-steep improvement) exhibited some evidence of improvement in working memory and PTSD symptoms, progress was overall modest. Higher baseline cognitive performance has also been linked to reduced cognitive improvement in other cognitive training studies (Matysiak, Kroemeke, & Brzezicka, 2019; Rahe et al., 2015; van der Donk et al., 2020), perhaps reflecting an EF ceiling effect that impedes more substantial improvement. Conversely, although the low-WM/no improvement subgroup exhibited low EF at baseline, they exhibited greater engagement of the treatment target on a neural level compared with the low-WM/steep improvement (i.e. relatively higher activity in working memory-related regions). That this subgroup showed greater activity than the low-WM/steep improvement group in areas underlying working memory but still exhibited similarly poor working memory performance could indicate that they had already reached their full working memory potential, thereby explaining their subsequent lack of improvement during WMT. However, it is also possible that the lack of improvement in this group stemmed from other neural differences that we did not measure in this study. These differences could include differences in functional connectivity, either within working memory areas or between working memory and nonworking memory-related areas, or differences in neural activation involving areas that were not included in our analysis, either because they were too small or because they were outside of our predefined working memory mask.

Importantly, subgroups that exhibited greater WM improvement also tended to show more improvement on PTSD symptoms, echoing findings of prior training paradigms in which training-related enhancement of EF mediated improvement in PTSD symptoms (Badura-Brack et al., 2015). This finding suggests that enhancing working memory-related brain activity might improve top-down control of intrusive trauma-related memories and emotions (Aupperle et al., 2012). Indeed, improvement in top-down control has been linked to PTSD improvement in both traditional and novel PTSD treatments (Adenauer et al., 2011; Shaw et al., 2023). Furthermore, lower activity in frontal brain areas thought to be involved in both working memory and top-down control have been shown to predict greater PTSD symptom improvement among subgroups with low baseline EF (Nicholson et al., 2017). Accordingly, impoverished baseline activity in areas relevant to top-down control may afford the opportunity to improve engagement in those areas during training, facilitating a reduction in PTSD symptoms.

Clinically, our findings have at least two important implications. The first is that WMT could offer an alternative treatment option for PTSD if delivered to the right patients. The low-WM/steep improvement subgroup was estimated to experience a 12-point decline on the PCL-5, which is above the threshold for reliable change (Blanchard et al., 2023; Marx et al., 2022). Although this degree of symptom reduction is similar to gold standard PTSD therapies and was experienced by a smaller proportion of patients (~20% vs. ~50% in gold standard PTSD therapies) (Eftekhari et al., 2013), it was accomplished with a less time- and resource-intensive treatment and with a subgroup of patients (i.e. those with low EF) who tend not to respond to traditional PTSD therapies (Keyan et al., 2024). Thus, although further research is required, our findings preliminarily implicate WMT as a promising alternative for PTSD patients with low EF who might not otherwise respond to traditional therapies. The second implication is that successfully identifying patients who will respond to WMT may require both neuropsychological and neuroimaging assessments. At baseline, neuropsychological testing could not adequately distinguish the groups with the most divergent outcomes (i.e. low-WM/steep improvement and low-WM/no improvement subgroup), as they could only be differentiated when comparing their working memory-related brain activity. Although neuroimaging largely remains cost prohibitive for universal clinical use, a less costly and invasive tool (e.g. EEG) could potentially provide similar mechanistic clarity of poor EF performance while still allowing successful WMT candidates to be identified. Regardless, our results indicate that WMT may be best suited for those exhibiting deficits in working memory and/or EF prior to therapy, as about 50% of patients with low WM/EF scores demonstrated a strong therapeutic response. Future studies of this training protocol should consider restricting the study to PTSD patients exhibiting lower WM and/or EF scores.

The present study is not without limitations. First, our sample size was relatively small, particularly for neurocognitive outcomes and subgroup sizes. Second, PTSD symptoms were not assessed intensively during training, which precluded us from verifying that working memory changes facilitated PTSD changes (as opposed to the other way around). Of note, our training intervention involved repeated completion of a working memory task and contained no techniques that directly targeted other cognitive or behavioral mechanisms, making it more likely that working memory changes facilitated PTSD changes rather than vice versa. Third, our study did not include a control condition that involved an intervention other than WM training (e.g. a psychosocial intervention, a cognitive training targeting a different domain), so we cannot fully rule out the possibility that the low WM/steep improvement subgroup is a generally responsive group rather than specifically responsive to WM training. Poor baseline neurocognitive functioning typically predicts poorer responses to PTSD treatment (Crocker et al., 2018; Tanev et al., 2020; Van Praag et al., 2021), which contradicts the explanation that the low-WM/steep improvement subgroup would have the best response; however, the steep improvement in this group also suggests a higher propensity for learning and adaptation, which theoretically could make them more responsive to other treatment modalities (Cenkner et al., 2021). Thus, continued work is needed to determine whether the observed effects are global or specific to WM training. Relatedly, we cannot conclude that the steep changes observed in this group were due to the WM training versus elements shared by other interventions. While the lack of robust cognitive gains reported from other interventions deems this explanation less likely (Susanty et al., 2022), it nonetheless requires empirical investigation. Fourth, we did not have enough posttreatment EF data to investigate whether training-related improvements in working memory generalized to other EF domains, which should be verified in future studies. Finally, the difficulty of our WMT procedure was not adaptive. This lack of adaptability may have impeded the opportunity for improvement in the high-WM/shallow improvement group (i.e. by making the training more challenging) and the low-WM/no improvement group (i.e. by making the training less challenging). Notably, the low-WM/no improvement group had similar working at baseline as the low-WM/steep improvement group, suggesting that adapting the training based on initial working memory would not have made a difference. However, adaptation that continues throughout training could allow greater success for patients who are failing to improve, which should be a focus of future research into the effectiveness and prediction of WM training.

Despite these limitations, the present study still offers promising evidence that WMT could be a viable alternative for PTSD patients with low EF and that these patients might be identified using a combination of neuropsychological testing and neuroimaging. Future research should explore whether patients with these same neurocognitive and neural markers show poorer responses to traditional PTSD therapies and whether these markers can be used to successfully match PTSD patients to WMT in a prospective manner. Such work would help verify the clinical utility of WMT as a viable alternative for PTSD patients who tend not to respond to traditional therapies.

## Supporting information

10.1017/S0033291726103298.sm001 Hunt et al. supplementary material Hunt et al. supplementary material

## Data Availability

Requests for de-identified data, analysis code, and research materials should be made to Jessica Bomyea, jbomyea@health.ucsd.edu.

## Supplementary material

The supplementary material for this article can be found at http://doi.org/10.1017/S0033291726103298.
