# Supplementary material for: Pretraining neural and neuropsychological measures of executive functioning are associated with response to working memory training in Veterans with PTSD
Source: Psychol Med. 2026 Mar 2;56:e57. doi: 10.1017/S0033291726103298 (PMC12969206; doi:10.1017/S0033291726103298)
Supplement: Hunt et al. supplementary material [file S0033291726103298sup001.docx]

**Veterans who Respond Best to Working Memory Training for PTSD Exhibit Lower Executive Functioning and Working Memory-Related Brain Activity at Baseline**

Christopher Hunt^a^, Morgan M. Caudle^b^, Amy Jak^a,c^, Alan Simmons^a,c^**,** Jessica Bomyea^a,c^

^a^VA San Diego Center of Excellence for Stress and Mental Health

^b^University of California, San Diego

^c^SDSU/UCSD Joint Doctoral Program in Clinical Psychology

***Supplemental Materials***

**2. Methods**

**2.1 Participants**

Participants were Veterans recruited from the VA San Diego Healthcare System. Veterans were recruited in response to 1) flyers posted in mental health and primary care clinics, and general community areas as authorized by the VA San Diego Healthcare System, 2) advertisements in print and web-based media in the general community, and 3) referrals from mental health and primary care clinics and research studies. Study inclusion criteria included 1) meeting primary current DSM-5 criteria for posttraumatic stress disorder, 2) ages 21 to 65, 3) literacy in English, 4) intention to stay in the San Diego area for the duration of the study for completing in-person visits, and 5) willingness to attend assessment and treatment sessions. Exclusion criteria for the study included 1) lifetime history of psychotic disorders or bipolar disorder, 2) severe substance use disorder within the last year, 3) psychiatric conditions likely to adversely impact cognition and/or are deemed to require other primary psychological intervention, 4) a history of any neurological disorder that may be associated with cognitive dysfunction, including history of severe traumatic brain injury, 5) acute suicidality (defined as intent, plan and/or action for severe self-harm within the past 3 months), 6) presence of circumstances that required imminent intervention prior to other treatment (e.g., current domestic abuse), 7) plans for medication changes during the study timeframe, 8) current or planned evidence-based psychotherapy for PTSD within the study timeframe, 9) plans for changes to other psychosocial therapy within the study timeframe, and 10) presence of life-threatening or unstable medical conditions. Veterans who were taking psychotropic medications and followed VA standard of care medication guidelines and those receiving psychosocial treatment for non-PTSD conditions were included in this study as long as they met a 6-week stability criterion.

Of the 130 participants who were initially recruited for the study, 47 dropped did not meet eligibility criteria, two failed to initiate treatment, and two failed to attend the two-session minimum necessary to be included in analyses of WMT treatment trajectories, leaving a final analyzable sample of N = 79. Additionally, 30 of these 79 participants did not complete the baseline fMRI reading span task scan because they did not attend or because they were recruited during the COVID-19 pandemic when in-person visits were restricted, while a further four participants were excluded due to quality control issues (e.g., low signal) resulting in a somewhat smaller (*n* = 45) sample available for fMRI analyses.

**2.2 Procedure**

Veterans who were interested in the study completed a telephone screen to assess initial eligibility based on inclusion and exclusion criteria. All participants completed a baseline intake appointment where they provided informed written consent and initial clinical interviews and self-report measures. The Clinician Administered PTSD Scale for DSM-5 (CAPS-5), the Mini-International Neuropsychiatric Interview (Sheehan et al., 1998), and interview questions regarding treatment and medical history were used to evaluate final eligibility. The subset of participants deemed eligible and safe to complete magnetic resonance imaging (MRI) also completed a scan visit before and after training.

Following completion of baseline assessments, participants were randomized 1:1 to 16 sessions of high interference control WMT (HIC) or low interference control WMT (LIC). Randomization followed a randomly permuted block design and stratified by concurrent treatment status (none, medication, psychotherapy for non-PTSD conditions, or both) and age (dichotomized <36 or >=36). Treatment assignment was blinded for the PI, research staff administering assessments and training, and participants. Both HIC and LIC included 16 sessions completed over eight weeks (twice weekly). The HIC and LIC conditions differed only on the amount of interference control required to successfully remember stimuli on each trial. Participants randomized to HIC completed trials that required high interference control, where all to-be-remembered items were derived from the same category (e.g., all letter stimuli). Participant randomized to LIC completed trials that required less interference control but the same amount of overall working memory capacity, where all to-be-remembered items alternated categories (e.g., letters, then numbers). Notably, differences between HIC and LIC conditions were not the focus of the current investigation and the groups were collapsed for all main analyses. Additional information on differences between HIC and LIC will be presented in the main outcomes paper (Bomyea et al., In Preparation).

A brief set of self-report questionnaires, including an assessment of PTSD symptoms, was conducted midway through treatment (i.e., following session 8). After treatment, participants completed post-training assessment visits where they repeated the pre-training assessments and completed a brief interview to assess changes to psychotherapy or medication during the study protocol. All participants provided written informed consent and the trial protocol was approved by the VA San Diego Institutional Review Board.

**2.3 Measures**

***2.3.1 Reading Span (R-SPAN) – Training Version.*** Working memory changes across WMT were assessed using the modified reading span task (R-SPAN) participants completed as part of the WMT intervention (see previous section for full description of task). Total correct on the task was calculated based on the number of items recalled correctly in the accurate serial order. Within each session, correct responses were separately tabulated for each of the three runs (range per run = 0 – 60), and the highest R-SPAN total served as the participant’s score for that session. Thus, R-SPAN scores putatively reflect the participant’s maximum working memory ability for each session. Additionally, blocks in which participants failed the secondary processing task – defined as answering less than 75% of the items correctly – were excluded. This was done to ensure that scores were not inflated because of participants rehearsing the to-be-remembered items during the period they were supposed to be focusing on the secondary processing task.

***2.3.2. Reading Span (R-SPAN) – Scanner Version.*** At baseline, participants also completed a standard R-SPAN task in the fMRI scanner (Bomyea, Taylor, Spadoni, & Simmons, 2018), which was used to assess initial working memory capacity prior to beginning WMT. The scanner R-SPAN task was identical to the treatment R-SPAN except that it included sets of 2, 4, or 6 letters or numbers, with each set size being repeated 7 times in pseudorandom order. As in the R-SPAN training version, we excluded scores in cases where participants failed the secondary processing task. However, the criterion for exclusion was relaxed slightly for the baseline version – 50% correct rather than 75% for – given that participants were naïve to the task at baseline and thus more likely to fail because of unfamiliarity rather than rehearsal of to-be-remembered items and the increased difficulty of completing the in-scanner task due to the use of a joystick while lying down in the scanner.

***2.3.3 Symmetry Span (S-SPAN).*** At baseline, participants also completed a symmetry span (S-SPAN) task to assess initial working memory capacity in a modality different than what was trained during treatment (i.e., visuospatial rather than verbal working memory) (Oswald et al., 2014). During the S-SPAN task participants were instructed to remember the location of a red square while simultaneously deciding if a matrix was symmetrical. A black and white 8x8 matrix appeared on the screen, then after indicating that they had finished viewing the matrix via a button press, participants indicated whether the matrix was symmetrical or asymmetrical along a vertical line. After the participant finished the symmetry portion, a to-be-remembered red square, positioned in a 4x4 matrix, appeared on the screen for 650 ms. After all symmetry problems and red squares were presented for the trial, the participant viewed a recall screen where they selected the position and serial order of the red squares presented during the trial using the cursor. After the recall phase for the set was completed, the next trial began. The S-SPAN task consisted of 14 symmetry judgements, and set sizes of two to five to-be-remembered locations. S-SPAN score was calculated by summing the proportion of all correctly recalled items in each trial.

***2.3.4. Delis-Kaplan Executive Functioning System (D-KEFS; Delis et al., 2001)*.** At baseline, participants also completed two subtests from the D-KEFS: the Trail Making Test (TMT) and the Color-Word Interference (CWI). Each subtest provided a measure of EF, which allowed to examine EF differences between classes at baseline, as well as a measure of processing speed, which helped establish whether classes differed specifically on EF differences or on cognition more generally (i.e., differences in both EF and processing speed). The TMT processing speed measure was the raw score on the number sequencing portion while the EF measure was the raw score from the letter-number switching portion. The CWI processing speed measure was the raw score from word naming portion while the EF measure was the raw score from the inhibition-switching portion. Raw scores were used in place of scaled scores in order to maximize variance. Moreover, classes were did not differ on the basis of age and education (see Table 3 of main text), indicating that age- and education-based scaled score corrections were unnecessary for the current study. Each raw scores reflects the total time needed to complete the given exercise; thus, lower scores indicate better performance.

***2.3.4. The PTSD Checklist for DSM-5 (PCL-5).*** The PTSD Checklist for DSM-5 (PCL-5; Weathers et al., 2013) is a 20-item self-report measure of PTSD symptom severity. Respondents rate how bothersome each symptom is from 0 (not at all) to 4 (extremely bothersome). The PCL-5 was given at pre-treatment, mid-treatment, and post-treatment. Scores range from 0 – 80; higher scores reflect greater PTSD symptom severity.

***2.3.5. Insomnia Severity Index (ISI).*** The Insomnia Severity Index (ISI; Morin, 1993) is a 7-item self-report measure of insomnia symptoms. Veterans responded with a 0 (None) to 4 (Very Severe) scale on their difficulty falling asleep, staying asleep, and waking up too early. The remaining questions queried satisfaction with sleep pattern, effect of sleep on daytime and social functioning, and concerns about current sleep difficulties on the same 0 to 4 scale. Scores range from 0 – 28; higher scores indicate more severe insomnia symptoms.

***2.3.6. Beck Depression Inventory – II (BDI-II).*** The BDI-II (Beck et al., 1996) is a 21-item self-report measure used to evaluate depressive symptoms according to the DSM-IV. Subjects rate the severity of their depressive symptoms in the past two weeks on a 4-point Likert-type scale from 0 – 3. Scores range from 0 – 63; higher scores reflect greater depression severity.

***2.3.7 Traumatic Brain Injury Screen.*** Participants were also asked to self-report history related to prior head injuries in order to assess possible presence of traumatic brain injury (TBI). Specifically, participants were asked whether they had ever sustained a prior head injury or concussion as well as loss of consciousness, post-traumatic amnesia, and disorientation/confusion occurred as a result of the injury. Based on their responses, participants were classified into the TBI severity categories (0 = “none”, 1 = “mild”, 2 = “moderate”, 3 = “Severe”) using established TBI diagnostic guidelines (O’Neil & Storzbach et al., 2013).

2.3.8 Clinician-Administered PTSD Scale for DSM-5 (CAPS-5). The semi-structured CAPS-5 interview was used to assess severity of PTSD symptoms and to determine PTSD diagnostic status (Weathers et al., 2018). PTSD diagnostic status is determined based on the presence of the minimum number of symptoms in each PTSD criterion. A symptom is considered present only if the severity of the symptoms is rated as moderate or more severe.

2.3.9 Mini-International Neuropsychiatric Interview (MINI). Participants completed the semi-structured MINI interview which assesses the presence of various mental health disorders according to the DSM-5 criteria (Sheehan et al., 1998).

**2.4 Statistical analyses**

Analyses for the current study are organized into four sections: 1) Class identification, which utilized session-by-session R-SPAN training task to identify latent WMT classes; 2) class validation, which whether the extracted WMT classes exhibited different patterns of change on two exogenous outcomes (spatial working memory, PTSD symptoms); and 3) characterization, which involved comparing classes on baseline demographic, neurocognitive, and neural variables to better determine whether classes could be readily differentiated prior to training.

***2.4.1 WMT class identification.*** Identification of latent WMT classes was accomplished using growth mixture modeling (GMM), a statistical technique used to identify un-observed subsamples in a dataset based on differences in longitudinal change on one more variables of interest (Ram & Grimm, 2009). Each GMM was run using the hlme command within the lcmm package in R (Proust-Lima et al., 2015). Specifically, we used the ‘gridsearch’ subcommand to run each GMM with 100 iterations. This function permits estimation of a model from a random set of initial values, which is necessary guard against obtaining biased parameter estimates that represent local rather than absolute maxima (Biernacki et al., 2003). The function then automatically retains estimates from the best-fitting model (i.e., lowest log likelihood) after reaching the max number of iterations.

In all models, session-by-session R-SPAN peak scores (see previous section for calculation) served as the dependent variable while session number (1 – 16) served as the within-subject predictor variable. Model comparison was carried out by comparing model fit indices across candidate models, which were evaluated in two steps. First, we sought to identify the optimal 1-class model (hereby referred to as the base model), among models with different combinations of random effects (none, intercept, or intercept and slope) and slope shapes (linear and logarithmic). Testing of logarithmic effects allowed us to test whether working memory improved more rapidly nearer the beginning of WMT relative to the end, which is commonly the pattern of response observed in mental health treatments (Hayes et al., 2007). Once the best-fitting base model was identified, it was re-tested with additional latent classes added until model fit was no longer improved. The model with this optimal number of latent classes was then selected for interpretation of class-specific intercepts and slopes. Model fit was assessed through examination of Bayesian Information Criterion (BIC), with lower values indicating better model fit.

***2.4.2 WMT Class Validation.*** The extracted WMT classes were then validated by examining whether they showed a similar pattern of differences on two exogenous variables: spatial working memory (S-SPAN) and PTSD symptoms (PCL-5). Differences between classes on S-SPAN changes were examined because S-SPAN is a working memory task from a different domain than the training task (visuo-spatial rather than verbal) and could thus help verify that classes showed different patterns of working memory change and not just different patterns of change on the training task. Differences in PCL-5 changes were tested to examine whether observed differences in working memory changes between classes translated into differences in PTSD symptom changes across training. For each outcome, we tested a mixed effects model (MEM) to determine whether the extracted WMT classes were related to changes in S-SPAN scores across training. In each model, time served as the within-subject fixed effect and was coded as 0 (pretreatment), 1 (mid-treatment), and 2 (post-treatment). Since S-SPAN was not measured at mid-treatment, its model only included the pre- and post-treatment timepoints. For each outcome, We first identified the best-fitting base model by testing different combinations of random effects (intercept or intercept and slope) and slope shape (linear versus logarithimic), selecting the model with the lowest BIC value for interpretation. Since the S-SPAN model only included two time points, logarithmic models were not tested, as they require a minimum of three timepoints. Then, within the best-fitting model for each outcome, we added WMT class and the Time x WMT class interaction as additional fixed effects. Here, a significant WMT class x Time interaction reflects a significant difference between classes in the degree of change in the outcome (S-SPAN or PCL-5). Since the WMT class was a categorical variable, we utilized contrast coding to evaluate its effect, wherein the WMT class with the most positive slope (i.e., greatest improvement in peak R-SPAN scores across training) was treated as the reference group that was compared to the other classes.

***2.4.3 Class characterization.***

***2.4.3.1 Self-reported and Neurocognitive data.*** Next, we compared the extracted WMT classes on key covariates that might putatively relate to differences in working memory according to past research, namely: Age, education level, sleep (ISI total), PTSD symptoms (PCL-5 total), time since trauma (in months) depression symptoms (BDI-II total), and TBI status. We also compared participants on neurocognitive variables related to executive functioning, namely R-SPAN, S-SPAN, D-KEFS TMT sequencing, and D-KEFS CWI inhibition/switching, as well as two neurocognitive measures of processing speed (D-KEFS TMT visual scanning, D-KEFS CWI word reading) to assess if differences were related specifically to EF or were observed across cognition more broadly. As a validity check, we also examined differences in the proportion of participants assigned to the HIC versus LIC groups to verify that the WMT cluster solution wasn’t merely a recapitulation of treatment groups, and in the number of training sessions attended to check whether differences in response to training were due to differences in training engagement. Comparisons of continuous variables were carried out using a one-way ANOVA in which class membership served as the independent variable and the covariate as the dependent variable. For all comparisons, we applied a benjamini-hochberg correction for eight multiple comparisons, which involves ranking observed p-values from lowest to highest and evaluating them according to the formula (I/M)*Q where I is the test’s rank, M is the number of tests, and Q is the original significance level (i.e., .05, two-tailed). Significant effects of omnibus comparisons were followed-up with post-hoc pairwise comparisons, again applying a Benjamini-Hochberg correction for the number of pairwise comparisons.

***2.4.3.2. Neural data.*** The fMRI data processing pipeline was conducted utilizing Analysis of Functional NeuroImages (AFNI)(v23.1.05)(Cox, 1996) and Advanced Normalization Tools Software in R (ANTsR_0.5.7.4)(Avants et al., 2011). The fMRI preprocessing steps performed on all imaging data included slice time correction (AFNI:3dTshift), temporal outlier removal (AFNI:3dDespike), field inhomogeneity correction (ANTsR:abpN4), and removal of regressors identified during motion correction and CompCor estimation. To facilitate group-level analysis, individual data were aligned using individual anatomical and MNI templates (ANTsR:antsRegistration/antsApplyTransforms).

To analyze neural functioning during the R-SPAN task, response regressors were created for each phase of the task. Three phases were determined by the unique timing of presentation for each phase for each participant, including sentence reading and verification, stimuli encoding, and stimuli recall. The encoding and recall phases were each weighted by set size to model working memory load variance and weighted by proactive interference (PI) control demand (Hamilton et al., 2022; Bomyea et al., 2015) to reflect the cumulative increase in PI with consecutive presentation of similar-to-be remembered items.

To investigate differences in neural activation during working memory across WMT class differences, a 3dLME (AFNI 3dLME) was performed within an a-priori defined working memory mask using neuroanatomical atlases of regions relevant to working memory (NeuroSynth v5 “working memory “ Topic 045). Primary analyses used a generalized linear test compared activation between WMT classes, WMT non-responders versus responders, during the encoding phase of the task, weighted according to set size (i.e., the number of stimuli presented in a single trial). A supplemental analysis used a planned contrast to compare activation across the three WMT classes during the encoding phase to determine potential neural differences between the WM/steep improvement and the other low-improving groups. To control for multiple corrections within the mask (voxel-wise a-prior probability of p< .001, corrected cluster-wise activation probability of 0.05), we utilized AFNI’s updated 3dClustSim program to conduct permutation testing (Boynton et al., 1996; Cox et al., 2016). Significant activations with at least 10 contiguous voxels within the working memory mask were considered.

***2.4.3.3 Evaluating clinical significance of baseline measures***

Finally, to evaluate the clinical significance of baseline variables that successfully differentiated WMT classes, we entered each variable as a predictor in a separate mixed effects model predicting PCL-5 changes across training. This analysis was done to verify the prospective value of these baseline variables for identifying patients who would exhibit a clinical response to WMT as indicated by a decrease in PTSD symptoms across training.

WMT class identification using GMM was performed in RStudio version 2022.02.3. Covariate comparisons and mixed effects models were performed in SPSS version 28. Alpha was set at .05 (two-tailed) for all statistical tests, unless otherwise noted.

**3. Results**

**3.1 WMT class identification**

For the base model, the model with a random intercept, random slope, and a logarithmic slope shape provided the best fit among the six candidate models. Addition of latent classes to this model revealed that the three-class solution provided the optimal fit, as the four-class solution resulted in an increase in BIC, indicating poorer fit when penalized for less parsimony. The full set of model comparison statistics can be found in Table 2S.

When repeating the GMM only including participants who completed the baseline neuropsychological assessment, the best-fitting model remained the same (i.e., random slope and intercept, logarithmic slope shape, three latent classes; see Table 2S for full model comparison statistics). Moreover, the latent class parameters of the best-fitting model for the restricted sample were essentially the same as those in the full sample. Specifically, the three latent subgroups included: 1) a high-WM/no improvement subgroup (50.91% the total sample), characterized by a high intercept (*b* = 52.26 95% CI = [50.36, 54.16], *p* < .001) and shallow but significant positive slope (*b* = 1.67, 95% CI = [0.97, 2.37), *p* <. 001); 2) a low-steep improvement subgroup (21.8% of sample), characterized by a low intercept (*b* = 29.86, 95% CI = [23.30, 36.42], *p* < .001) and a steep positive slope (*b* = 7.76, 95% CI = [6.00, 9.52], *p* < .001); and 3) and a low-WM/no improvement subgroup (27.27% of the sample) that was also characterized by a low intercept (*b* = 32.92, 95% CI = [23.18, 42.66], *p* < .001) but exhibited a nonsignificant negative slope (b = -1.47, 95% CI = [-3.51, 0.57], *p* = .150)

**3.2 WMT Class Validation**

Results of model fitting procedures for both S-SPAN and PCL-5 can be found in Table 2S. Both outcomes were best modeled with a random intercept (but not a random slope). The PCL-5 model exhibited a slightly better fit for the logarithmic model and was therefore modeled accordingly.

**3.3 WMT Class Characterization**

***3.3.1 Self-report and neuropsychological data.*** Post-hoc comparisons revealed that the high-WM/no improvement subgroup exhibited higher baseline scores on the R-SPAN relative to low-steep improvement subgroup (*M*_diff_ *=* 20.77*, t*(56) *=* 5.38, *p* < .001) and the Low-WM/no improvement subgroup (*M*_diff_ *=* 26.50*, t*(56) *=* 6.93*, p* < .001 ), on the S-SPAN relative to low-steep improvement subgroup (*M*_diff_ *=* 8.61*, t*(56) *=*3.70 *, p* <.001) and the Low-WM/no improvement subgroup (*M*_diff_ = 11.27 *, t*(60) = 6.62, *p* <.001), on D-KEFS TMT switching relative to the Low-WM/no improvement subgroup (*M*_diff_ *=* -34.75*, t*(41) *=* -3.17*, p* = .006), and on D-KEFS CWI inhibition/switching relative to the low-steep improvement subgroup (*M*_diff_ *=* -17.68*, t*(40) *=* -3.16*, p* = .007) and the low-WM/no improvement subgroup (*M*_diff_ *=* -23.62*, t*(42) *=* -2.89*, p* = .012). Differences between the high-WM/no improvement and low-steep improvement subgroups on D-KEFS TMT were significant at the level of a trend favoring higher scores in the high-WM/no improvement subgroup (*M*_diff_ *=* -11.97*, t*(40) *=* -1.74*, p* = .090). Conversely, the low-WM/no improvement and low-steep improvements subgroups showed no differences on these same measures at baseline, including on R-SPAN (*M*_diff_ *=* -5.73, *t*(28) = -1.05, *p*  = .304), S-SPAN (*M*_diff_ *=* 2.66*, t*(32) *=* -1.27*, p* = .213), D-KEFS TMT Switching (*M*_diff_ *=* 22.78*, t*(23) *=* 1.85*, p* = .078), and D-KEFS CWI Switching/Inhibition (*M*_diff_ *=* 5.94*, t*(24) *=* 0.62*, p* = .541). There were no group differences in self-reported symptoms, time since trauma, number of training sessions attended, or proportion of group assigned to the active condition (*p*s > .030).

***3.3.2 Neural data.*** In a two-group analysis comparing the two groups with low baseline performance, the low-steep improvement class exhibited significantly lower neural activity than the Low-WM/no improvement class during Rspan encoding in the right middle frontal gyrus (MFG; *F*(2, 21) = 14.49, *p* = .001), the left precentral gyrus (PCG; *F*(2, 21) = 11.02) *p* = 0.004), the left PCG extending into the left MFG (*F*(2, 21) = 11.56, *p* = 0.003), the left MFG (*F*(2, 21) = 21.76, *p* < .001), and left inferior frontal gyrus (IFG; *F*(2, 21) = 13.55, *p* = .002; see Figure 3 for pictures of regions and Table 5S for coordinates). A three-group analysis revealed 13 total ROIs in which the low improvement subgroups (low-WM/no improvement class and the high-WM/no improvement) differed significantly from the low-steep improvement subgroup (see Table 6S for full list and coordinates). Five of the seven largest ROIs overlapped with those found in the two-group analysis, specifically the right MFG, *F*(2, 44) = 7.90, *p* = 0.001, left PCG, *F*(2, 44) = 7.23, *p* = 0.002, left PCG extending into the left MFG, *F*(2, 44) = 6.23, *p* = 0.004, left IFG, *F*(2, 44) = 7.77, *p* = 0.001, and left MFG, *F*(2, 44) = 9.61, *p* < 0.001. For each of these ROIs, pairwise comparisons revealed that the low-steep improvement group showed lower activation than the high-WM/no improvement group and the low-WM/no improvement groups, who did not differ from each other (*p*s < .018; see Figure 3S). The two of the seven largest ROIs not overlapping with the two-group analysis included the left supplementary motor area (SMA; *F*(2, 44) = 7.90, *p* = 0.001) and the right IPL/angular gyrus *F*(2, 44) = 4.13, *p* = 0.023). Pairwise comparisons for the right IPL/angular gyrus showed the same pattern as the other ROIs that overlapped with the two-group, wherein the low steep-improvement group shower lower activation than the other two groups (*p*s < .018), who did not differ from each other. In contrast, for the left SMA, the low-WM/steep improvement subgroup differed significantly from the high-WM/shallow improvement subgroup (*p* = .040), but not from the low-WM/no improvement subgroup (*p* = .177).

**Table 1S**

*Demographics and baseline clinical characteristics of the final sample*

| ***Variable*** | ***n (%)*** |
| --- | --- |
| **Gender** |  |
| *% Male* | 53 (67.1) |
| *% Female* | 26 (32.9) |
| **Race/Ethnicity** |  |
| *%Black/African American* | 7 (8.9) |
| *% White/Caucasian* | 48 (60.8) |
| *% Hispanic/Latino* | 6 (7.6) |
| *% Asian American* | 4 (5.1) |
| *% Native American/Alaskan Native* | 5 (6.3) |
| *% Other* | 3 (3.8) |
| *% Native Hawaiian/Pacific Islander* | 2 (2.5) |
| *% Unknown/Declined to Respond* | 4 (5.1) |
| **Education** |  |
| % Did not finish high school | 0 (0.0) |
| *% High School or GED Equivalent* | 11 (14.1) |
| *% Some Post High School but no Degree of Certificate* | 1 (1.3) |
| *% Post High School Degree or Certificate* | 7 (9.0) |
| *% 2-Year Associate Degree* | 22 (28.2) |
| *% 4-Year College Degree* | 17 (21.8) |
| *% Graduate School Degree* | 20 (25.6) |
| **Variable** | **Mean (SD)** |
| TBI Severity | 0.92 (0.76) |
| Mean Age (SD) | 38.27 (8.70) |
| Mean PCL-5 (SD) | 46.01 (15.01) |
| Mean BDI-II (SD) | 26.16 (10.89) |
| Mean ISI (SD) | 15.96 (5.84) |
| Mean R-SPAN Total (SD) | 54.12 (17.66) |
| Mean S-SPAN Total (SD) | 23.34 (9.01) |

*Note.* TBI severity was based on a questionnaire of worst TBI experienced and scored from 0 (no TBI) to 3 (severe TBI). GED = General education degree; TBI = Traumatic brain injury; PCL-5 = PTSD Checklist for DSM-5; BDI-II = Beck Depression Inventory – II; ISI = Insomnia Severity Index; R-SPAN = Reading span task; S-SPAN = Symmetry span task; SD = Standard deviation. *N* = 79.

**Table 2S.** Model fit indices of candidate growth mixture models.

| **Sample** | **Model** | **Random effects** | **Slope shape** | **# of classes** | **BIC value** |
| --- | --- | --- | --- | --- | --- |
| Full | 1 | None | Linear | 1 | 8213.93 |
| Full | 2 | None | Log | 1 | 8212.61 |
| Full | 3 | Intercept | Linear | 1 | 6544.37 |
| Full | 4 | Intercept | Log | 1 | 6525.53 |
| Full | 5 | Intercept + slope | Linear | 1 | 6442.05 |
| Full | 6 | Intercept + slope | Log | 1 | 6414.87 |
| Full | 6.1 | Intercept + slope | Log | 2 | 6356.63 |
| **Full** | **6.2** | **Intercept + slope** | **Log** | **3** | **6321.79** |
| Full | 6.3 | Intercept + slope | Log | 4 | 6323.81 |
| Neuropsych Only | 1 | None | Linear | 1 | 5587.85 |
| Neuropsych Only | 2 | None | Log | 1 | 5587.98 |
| Neuropsych Only | 3 | Intercept | Linear | 1 | 4487.90 |
| Neuropsych Only | 4 | Intercept | Log | 1 | 4474.26 |
| Neuropsych Only | 5 | Intercept + slope | Linear | 1 | 4387.52 |
| Neuropsych Only | 6 | Intercept + slope | Log | 1 | 4366.42 |
| Neuropsych Only | 6.1 | Intercept + slope | Log | 2 | 4323.71 |
| Neuropsych Only | 6.2 | **Intercept + slope** | **Log** | **3** | 4306.73 |
| Neuropsych Only | 6.3 | Intercept + slope | Log | 4 | 4308.87 |

Note. The base model was selected as best-fitting among models 1 – 6. Models were first tested with only one latent class to determine which combination of random effects and slope shape afforded the best fit. Then, within the best-fitting base model (Model 6), the number of latent classes was added until there was no longer a decrease in BIC. Lower BIC values indicate better model fit. Full refers to models tested with the full sample (*n* = 79. Neuropsych Only refers to models tested including only those who completed the baseline neuropsychological assessment (*n* = 56). Best-fitting overall model that was selected for interpretation is shown in bold. BIC = Bayesian Information Criterion.

**Table 3S**

Fit statistics for unconditional models of S-SPAN and PCL-5 scores across WMT

| ***DV*** | ***Model*** | **BIC** | **Random Effect** | **σ^2^** | **p** | **Slope Type** | **b** | **SE** | **p** |
| --- | --- | --- | --- | --- | --- | --- | --- | --- | --- |
| S-SPAN | **1** | **966.34** | **Intercept** | **28.57** | **<.001** | **Linear** | **1.24** | **0.49** | **.015** |
|  | 2 | 981.21 | Intercept + Slope | 37.18 | <.001 | Linear | 1.12 | 0.66 | .090 |
| PCL-5 | 1  2 | 747.06  751.53 | Intercept  Intercept + Slope | 80.67  82.85 | <.001  <.001 | Linear  Linear | -2.93  -2.94 | 1.19  1.51 | .016  .054 |
|  | **3** | **746.30** | **Intercept** | **79.59** | **<.001** | **Log** | **-5.54** | **2.10** | **.011** |
|  | 4 | 750.70 | Intercept + Slope | 81.53 | <.001 | Log | -5.74 | 2.66 | .033 |

Note. PCL-5 was modeled across three timepoints (pre-, mid- and post-treatment) while S-SPAN was modeled across two (pre- and post-treatment). All models included time as the within-subject predictor variable. Because only two timepoints were available for S-SPAN, logarithmic slope models were not tested. Best-fitting overall model that was selected for interpretation for each outcome is shown in bold. Lower BIC values indicate better fit. S-SPAN = Symmetry Span; PCL-5 = PTSD Checklist for DSM-5; BIC = Bayesian Information Criterion; SE = Standard error; Log = Logarithmic.

**Table 4S**

*Results of mixed effects models predicting changes in spatial working memory, PTSD symptoms, and depression symptoms from WMT subgroups*

| **DV** | **Fixed Effect** | **B (95% CI)** | **t** | **p** |
| --- | --- | --- | --- | --- |
| S-SPAN | High-WM/no improvement subgroup | 11.61 (5.60, 17.62) | 3.82 | <.001 |
|  | Low-WM/no improvement subgroup | -0.04 (-7.10, 6.82) | -0.04 | .969 |
|  | Time | 3.56 (1.50, 5.62) | 3.46 | <.001 |
|  | High-WM/no improvement x Time | -2.83 (-5.22, -0.44) | -2.37 | .023 |
|  | Low-WM/no improvement x Time | -3.20 (-6.05, -0.35) | -2.24 | .029 |
|  |  |  |  |  |
| PCL-5 | High-WM/no improvement subgroup | 0.22 (-8.86, 9.29) | 0.05 | .962 |
| Total | Low-WM/no improvement subgroup | 9.17 (-1.21, 19.55) | 1.75 | .083 |
|  | Log time | -10.61 (-16.56, -4.66) | -3.53 | <.001 |
|  | High-WM/no improvement x Log time | 4.28 (-2.68, 11.23) | 1.22 | .226 |
|  | Low-WM/no improvement x Log time | 10.00 (1.77, 18.21) | 2.40 | .018 |
|  |  |  |  |  |
| PCL-5 | High-WM/no improvement subgroup | 0.01 (-2.48, 2.51) | 0.01 | .991 |
| Re-exp | Low-WM/no improvement subgroup | 2.86 (0.01, 5.72) | 1.99 | .049 |
|  | Log time | -2.84 (-4.67, -1.01) | -3.08 | .003 |
|  | High-WM/no improvement x Log time | 1.79 (-0.34, 3.92) | 1.66 | .099 |
|  | Low-WM/no improvement x Log time | 3.04 (0.52, 5.56) | 2.39 | .018 |
|  |  |  |  |  |
| PCL-5 | High-WM/no improvement subgroup | 0.62 (-0.96. 2.20) | 0.78 | .436 |
| Avoid | Low-WM/no improvement subgroup | 1.89 (0.08, 3.69) | 2.07 | .041 |
|  | Log time | -1.65 (-2.78, -0.52) | -2.89 | .005 |
|  | High-WM/no improvement x Log time | 0.74 (-0.58, 2.07) | 1.11 | .268 |
|  | Low-WM/no improvement x Log time | 1.03 (-0.53, 2.59) | 1.30 | .195 |
|  |  |  |  |  |
| PCL-5 | High-WM/no improvement subgroup | -0.98 (-4.47, 2.51) | -0.56 | .578 |
| Neg | Low-WM/no improvement subgroup | 1.53 (-2.46, 5.52) | 0.76 | .449 |
| Mood | Log time | -3.94 (-6.16, -1.72) | -3.51 | <.001 |
|  | High-WM/no improvement x Log time | 1.78 (-0.80, 4.38) | 1.37 | .175 |
|  | Low-WM/no improvement x Log time | 3.57 (0.50, 6.63) | 2.30 | .023 |
|  |  |  |  |  |
| PCL-5 | High-WM/no improvement subgroup | 0.54 (-2.31, 3.39) | 0.38 | .706 |
| Arousal | Low-WM/no improvement subgroup | 2.90 (-0.36. 6.16) | 1.76 | .081 |
|  | Log time | -2.17 (-4.12, -0.22) | -2.21 | .029 |
|  | High-WM/no improvement x Log time | 0.00 (-2.28, 2.28) | -.001 | .999 |
|  | Low-WM/no improvement x Log time | 2.37 (-0.32, 5.06) | 1.74 | .084 |
|  |  |  |  |  |
| BDI-II | High-WM/no improvement subgroup | -0.16 (-6.66, 6.32) | -0.05 | .960 |
|  | Low-WM/no improvement subgroup | 5.67 (-1.77, 13.11) | 1.51 | .134 |
|  | Log Time | -5.37 (-9,93, -0.91) | -2.38 | .019 |
|  | High-WM/no improvement x Log Time | 3.18 (-2.01, 8.38) | 1.21 | .228 |
|  | Low-WM/no improvement x Log Time | 4.82 (-1.43, 11.06) | 1.52 | .130 |

*Note*. All models were tested with a random intercept. Time was a 2-level within-subjects variable referring to pre- and post-WMT for S-SPAN and a 3-level within-subjects variable referring to pre-, mid-, and post-WMT for PCL-5. The WMT group variable was tested with contrast coding, wherein the low-steep improvement subgroup served as the reference group. All predictors reflect the effect relative to the reference group (responder group), with the exception of the time variables (Time and Log time), which reflect the effect in the reference group. The WMT groups were extracted from a GMM of R-SPAN peak changes across WMT. S-SPAN = Symmetry span; PCL-5 = PTSD Checklist for DSM-5; Re-exp = Re-experiencing subscale; Avoid = Avoidance subscale; Neg Mood = Negative mood and cognitions subscale; Arousal = Hyperarousal subscale; BDI-II. WMT = Working memory training; 95% CI = 95% Confidence interval.

**Table 5S**

*Regions of interest size and center of mass coordinates contrasting the low-WM/improvement class and the low-WM/steep improvement class*

| ROI | Voxels | x | y | z | *t* |
| --- | --- | --- | --- | --- | --- |
| Right MFG | 40 | 34 | 13 | 51 | 3.84 |
| Left PCG | 21 | -38 | -1 | 41 | 4.38 |
| Left PCG/MFG | 20 | -36 | 1 | 55 | 3.87 |
| Left MFG | 15 | -42 | 17 | 36 | 3.80 |
| Right IFG | 13 | 45 | 30 | 25 | 3.71 |
| *Note*. MFG = middle frontal gyrus; PCG = Precentral gyrus; IFG = Inferior frontal gyrus | | | | |  |

| Table 6S |  |  |  |  |  |
| --- | --- | --- | --- | --- | --- |
| *Regions of Interest size and center of mass coordinates contrasting the low-WM/no improvement class and the high-WM/shallow improvement class to the low-WM/steep improvement class* | | | | | |
| ROI | Voxel Size | *x* | *y* | *z* | *t* |
| Right MFG | 130 | 32 | 9 | 53 | 4.01 |
| Left PCG | 73 | -42 | 2 | 39 | 4.12 |
| Left PCG/MFG | 69 | -33 | 3 | 58 | 4.06 |
| Left SMA | 53 | -2 | 9 | 54 | 3.85 |
| Left IFG | 29 | -45 | 11 | 20 | 3.83 |
| Right IPL/ Angular gyrus | 27 | 35 | -56 | 39 | 3.88 |
| Left MFG | 23 | -44 | 17 | 36 | 4.05 |
| Right SMG | 21 | 47 | -40 | 38 | 3.9 |
| Left Cerebellum | 19 | -34 | -61 | -33 | 3.91 |
| Right IFG/MFG | 19 | 45 | 33 | 25 | 3.89 |
| Left MFG | 15 | -36 | 53 | 9 | 3.79 |
| Right IPL | 15 | 41 | -47 | 39 | 3.77 |
| Left IPL | 10 | -32 | -60 | 53 | 3.96 |
| *Note. MFG = Middle frontal gyrus; PCG = Precentral Gyrus; SMA = Supplementary motor area; IFG = Inferior frontal gyrus; IPL = Inferior parietal lobule; SMG = Supramarginal Gyrus* | | | | | |

**Table 7S**

*Results of mixed effects models predicting PCL-5 changes from baseline neural activity*

| **Fixed Effect** | **B (95% CI)** | **t** | **p** |
| --- | --- | --- | --- |
| Log Time | -8.47 (-13.11, -3.83) | -3.70 | <.001 |
| Right MFG | 5.95 (-0.43,12.34) | 1.90 | .066 |
| Log Time x Right MFG | 6.33 (1.89, 10.78) | 2.89 | .006 |
|  |  |  |  |
| Log Time | -10.77 (-15.50, -6.04) | -4.61 | <.001 |
| Left PCG | 1.39 (-0.04, 2.82) | 1.39 | .057 |
| Log Time x Left PCG2 | 1.54 (0.57, 2.52) | 3.21 | .003 |
|  |  |  |  |
| Log Time | -9.92 (-14.92, -4.92) | -4.02 | <.001 |
| Left PCG/MFG | 1.05 (-0.40, 2.49) | 1.47 | .151 |
| Log Time x Left PCG/MFG | 1.16 (0.13, 2.19) | 2.29 | .028 |
|  |  |  |  |
| Log Time | -6.18 (-11.11, -1.26) | -2.54 | .015 |
| Left MFG | 2.25 (1.03, 3.47) | 3.73 | <.001 |
| Log Time x Left MFG | 1.54 (0.41, 2.68) | 2.77 | .009 |
|  |  |  |  |
| Log Time | -10.07 (-15.62, -4.52) | -3.69 | <.001 |
| Right IFG | 1.28 (-0.11, 2.67) | 1.28 | .069 |
| Log Time x Right IFG | 1.13 (0.15, 2.12) | 2.33 | .026 |

*Note*. All models were tested with a random intercept and with a logarithmic slope for session. Session was a 3-level within-subject variable referring to baseline, mid-treatment, and post-treatment. WMT class included the low-WM/no improvement subgroup (coded as -1) and the low-WM/steep improvement subgroup (coded as 1), which were formed based on differences in changes in working memory scores across WMT. WMT = Working memory training. MFG = Middle frontal gyrus; PCG = Precentral gyrus; IFG = Inferior frontal gyrus

| **Table 8S** |  |  |  |  |  |  |
| --- | --- | --- | --- | --- | --- | --- |
| *Regions within a-priori defined working memory mask* | | | | |  |  |
|  | Center of Mass | |  | Peak | | |
| Voxels | x | y | z | x | y | z |
| 1505 | 39.4 | -12.3 | 36.2 | 50.4 | -7.2 | 13.2 |
| 1188 | 31.1 | 57.5 | 45.2 | 28.8 | 72 | 20.4 |
| 1177 | -16.6 | -10.2 | 50.9 | 0 | -24 | 34.8 |
| 1039 | -32.5 | 55 | 45.2 | -26.4 | 64.8 | 27.6 |
| 958 | -39.6 | -35.2 | 20.3 | -36 | -19.2 | -10.8 |
| 167 | -31.1 | 61.6 | -31.1 | -24 | 62.4 | -37.2 |
| 166 | 36.3 | -51.7 | 10.4 | 36 | -48 | 1.2 |
| 122 | 21.7 | 1.7 | -17.7 | 28.8 | 4.8 | -22.8 |
| 107 | -48.7 | -8.5 | 24.8 | -50.4 | -7.2 | 13.2 |
| 88 | 30 | -22.7 | 0.2 | 31.2 | -19.2 | -8.4 |
| 81 | 35.1 | 60.6 | -30.1 | 38.4 | 64.8 | -34.8 |
| *Note*. Mask defined using neuroanatomical atlases of regions relevant to working memory (NeuroSynth v5 “working memory” Topic 045). | | | | | | |

**Figure 1S.** CONSORT flow diagram summarizing participant progress throughout the study.

## Enrollment

Assessed for eligibility (n=130)

Included in main analysis (*n*=37)

♦ Available neuropsych data (n=26)

♦ Available neural data (n=21)

Included in main analysis (*n* = 42)

♦ Available neuropsych data (n=30)

♦ Available neural data (n=24)

## Analysis

Allocated to LIC (n=39)

♦ Completed < 2 WMT sessions (n=2)

♦ Unable to keep time commitment (n = 1)

♦ Lost computer access (n=1)

Allocated to HIC (n=42)

## Allocation

Excluded (n=49)

♦ Not meeting inclusion criteria (n=47)

No PTSD (n=12)

Psychosis or bipolar disorder (n=1)

No computer access (n=1)

AUD or SUD (n=3)

Ineligible concurrent treatment (n=6)

Medical exclusion (n=1)

Other (n=23)

♦ Lost prior to randomization (n=2)

Randomized (n=81)

**Figure 2S.** *Map of a-priori defined working memory mask.*


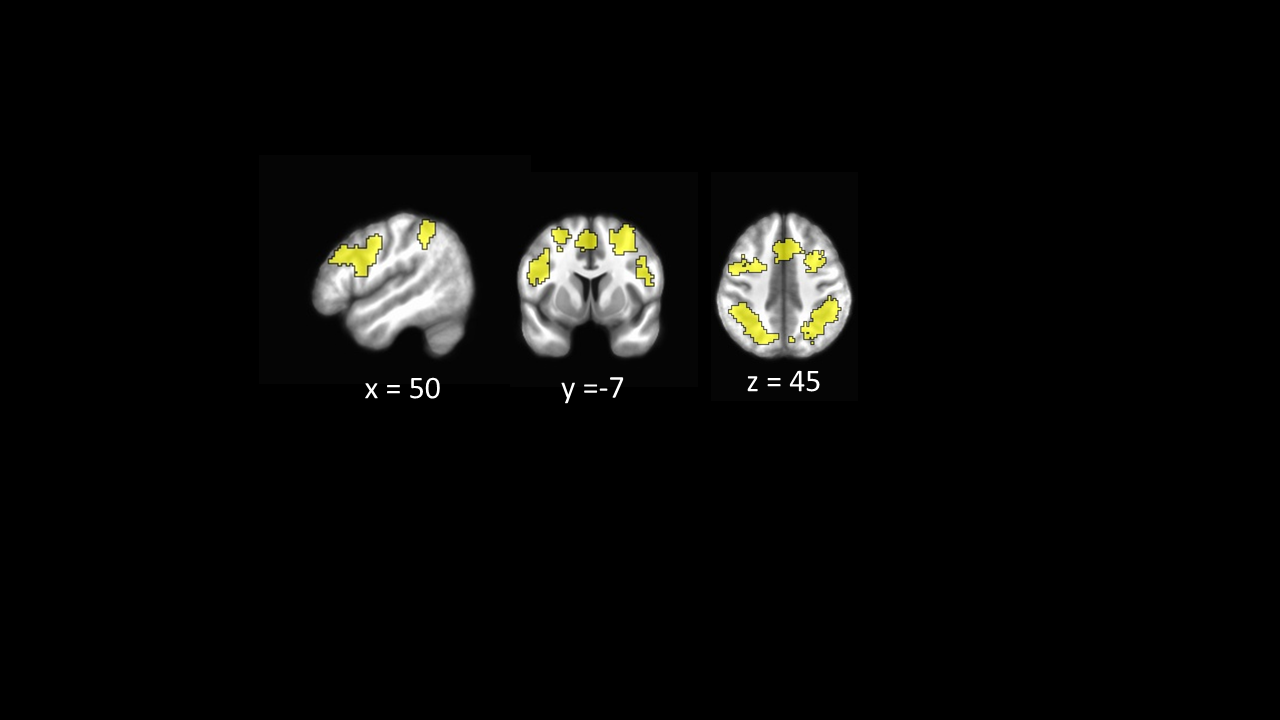


*Note*. Mask defined using neuroanatomical atlases of regions relevant to working memory (NeuroSynth v5 “working memory” Topic 045).

High-WM/Shallow-Improvement . Low-WM/No Improvement. Low-WM/Steep Improvement

**Figure 3S.** Regions of interest contrasting the low-WM/no improvement subgroup and high-WM/shallow improvement subgroup versus the low-WM/steep improvement subgroup that overlapped with regions contrasting the low-WM/no improvement and low-WM/steep improvement subgroups. *Note.* MFG = Middle frontal gyrus; PCG = Precentral Gyrus;; IFG = Inferior frontal gyrus. * *p* < .05; ** *p* < .01; *** *p* < .001.

**References**

Avants, B. B., Tustison, N. J., Song, G., Cook, P. A., Klein, A., & Gee, J. C. (2011). A reproducible evaluation of ANTs similarity metric performance in brain image registration. *Neuroimage, 54*, 2033-2044. doi:<https://doi.org/10.1016/j.neuroimage.2010.09.025>

Bomyea, J., Stein, M. B., & Lang, A. J. (2015). Interference control training for PTSD: A randomized controlled trial of a novel computer-based intervention. *Journal of Anxiety Disorders, 34*, 33-42. doi:10.1016/j.janxdis.2015.05.010

Bomyea, J., Taylor, C. T., Spadoni, A. D., & Simmons, A. N. (2018). Neural mechanisms of interference control in working memory capacity. *Hum Brain Mapp, 39*(2), 772-782. doi:10.1002/hbm.23881

Cox, R. W. (1996). AFNI: software for analysis and visualization of functional magnetic resonance neuroimages. *Comput. Biomed. Res., 29*, 162-173. doi:10.1006/cbmr.1996.0014

Sheehan, D. V., Janavs, J., Baker, R., Harnett-Sheehan, K., Knapp, E., Sheehan, M., . . . Lepine, J. P. (1998). MINI - Mini International Neuropsychiatric Interview - English Version 5.0.0 - DSM-IV. *Journal of Clinical Psychiatry, 59*, 34-57. doi:DOI 10.4088/JCP.09m05305whi

Weathers, F. W., Bovin, M. J., Lee, D. J., Sloan, D. M., Schnurr, P. P., Kaloupek, D. G., . . . Marx, B. P. (2018). The Clinician-Administered PTSD Scale for DSM-5 (CAPS-5): Development and initial psychometric evaluation in military veterans. *Psychol Assess, 30*(3), 383-395. doi:10.1037/pas0000486
